# Fruit development of the diploid kiwifruit, *Actinidia chinensis *'Hort16A'

**DOI:** 10.1186/1471-2229-11-182

**Published:** 2011-12-28

**Authors:** Annette C Richardson, Helen L Boldingh, Peter A McAtee, Kularajathevan Gunaseelan, Zhiwei Luo, Ross G Atkinson, Karine M David, Jeremy N Burdon, Robert J Schaffer

**Affiliations:** 1 The New Zealand Institute for Plant & Food Research Limited (PFR), PO Box 23, Kerikeri, 0245, New Zealand; 2 PFR Ruakura, Private Bag 3123, Hamilton, 3214, New Zealand; 3 PFR Mount Albert Private Bag 92169, Auckland, 1142, New Zealand; 4 The University of Auckland, School of Biological Sciences, Private Bag 92019 Auckland, 1142, New Zealand

## Abstract

**Background:**

With the advent of high throughput genomic tools, it is now possible to undertake detailed molecular studies of individual species outside traditional model organisms. Combined with a good understanding of physiological processes, these tools allow researchers to explore natural diversity, giving a better understanding of biological mechanisms. Here a detailed study of fruit development from anthesis through to fruit senescence is presented for a non-model organism, kiwifruit, *Actinidia chinensis *('Hort16A').

**Results:**

Consistent with previous studies, it was found that many aspects of fruit morphology, growth and development are similar to those of the model fruit tomato, except for a striking difference in fruit ripening progression. The early stages of fruit ripening occur as the fruit is still growing, and many ripening events are not associated with autocatalytic ethylene production (historically associated with respiratory climacteric). Autocatalytic ethylene is produced late in the ripening process as the fruit begins to senesce.

**Conclusion:**

By aligning *A. chinensis *fruit development to a phenological scale, this study provides a reference framework for subsequent physiological and genomic studies, and will allow cross comparison across fruit species, leading to a greater understanding of the diversity of fruits found across the plant kingdom.

## Background

The development of flower organs into fleshy fruits provides both efficient protection and dispersion of seeds. Fleshy fruits develop from swollen ovaries or other flower parts [1], with the structure laid down before or soon after flowering pollination and fertilisation [2]. Following fertilisation there is a period of rapid growth, facilitated initially by cell division that determines fruit shape, sink strength and size. Cell division may be completed 7-10 days after anthesis in tomato or extend up to 50 days after anthesis in orange [1]. Subsequent fruit growth is due to the expansion of cells modulated by seed development and its effect on fruit sink strength [3]. Towards the end of fruit growth, embryos mature and the fruit ripens, often exhibiting rapid changes in hormone concentrations, respiration, cell wall integrity, colour, aroma and flavour compounds [4]. These desirable characteristics have led to a long history of selection, commercial development and understanding of fruit crops like apple, grape, tomato, citrus and stone fruit. Many of these crops bear fruit with little resemblance to their wild relatives because of this long period of domestication. In contrast, all cultivated kiwifruit, including commercially important cultivars 'Hayward' (*Actinidia deliciosa *(A. Chev.) C.F. Liang et A.R. Ferguson), and 'Hort16A' (*Actinidia chinensis *Planch. var. *chinensis *'Hort16A') are only one or two generations removed from their wild relatives [5].

*Actinidia *species (family Actinidiaceae) share a number of common characters; they are all dioecious, with the ovary of the female flower formed by the fusion of many carpels with a whorl of free, radiating styles. The fruit is a berry containing many seeds in a juicy flesh [6]. Early research focused on cultivars within the hexaploid, green-fleshed, *A. deliciosa *kiwifruit [7-11]. However, because of its high ploidy number, molecular studies on this fruit are challenging and researchers are selecting the diploid genotypes of *A. chinensis *to understand the molecular processes of this genus. There is now a comprehensive genetic map of the 29 chromosomes of *A. chinensis *[12], a considerable number of ESTs [13], and it is readily transformable [14]. Finally, *A. chinensis *is currently the focus of an on-going genome sequencing programme (R Hellens, pers. comm.). Studies of fruit development in *A. Chinensis *'Hort16A' have focused on some aspects of fruit growth and colour development [15-19], while seasonal changes in fruit carbohydrate concentrations have been described for other *A. chinensis *genotypes [20]. One of the unusual features of *Actinidia *species is in their ripening behaviour, although classified as a climacteric fruit [11] the majority of ripening occurs before autocatalytic ethylene is produced [21].

The researchers of many plant species have benefitted from standardised descriptors of development, as this allows research studies to be compared under different environments or management systems to assess the effect of mutagenesis or specific transgenes. The most commonly used method is the Biologische Bundesantalt, Bundessortenamt und Chemische Industrie (BBCH) scale, which describes phenological changes in plant growth using a numeric scale with two decimal digits, the first to represent the principal stages (from 0 to 9) and second to represent secondary growth stages (from 0 to 9) [22]. BBCH scales for full plant growth are now available for many plants including *Arabidopsis thaliana *(converted to a 0-9.9 scale) [23], cereals [22], vegetable crops [24], pome and stone fruits [25], grapevine [26], and citrus [27]. While the BBCH scale has been used to describe the development of *A. deliciosa *'Hayward' [28], that study was not focused on fruit development and there was little detail of the physiological processes at each developmental step.

Here we present a detailed study of fruit development from anthesis through to fruit senescence for *A. chinensis *'Hort16A' [29]. Aspects of development studied include fruit growth and ripening (softening, flesh colour change and soluble solids accumulation), carbohydrate and acid accumulation, ethylene metabolism and gene expression. From this study, we propose a systematic model based on the BBCH system for describing the physiological development of the fruit that can be used as anchor points for subsequent kiwifruit development research.

## Results

### A BBCH scale for *A. chinensis *'Hort16A' fruit development

**Figure 1:**
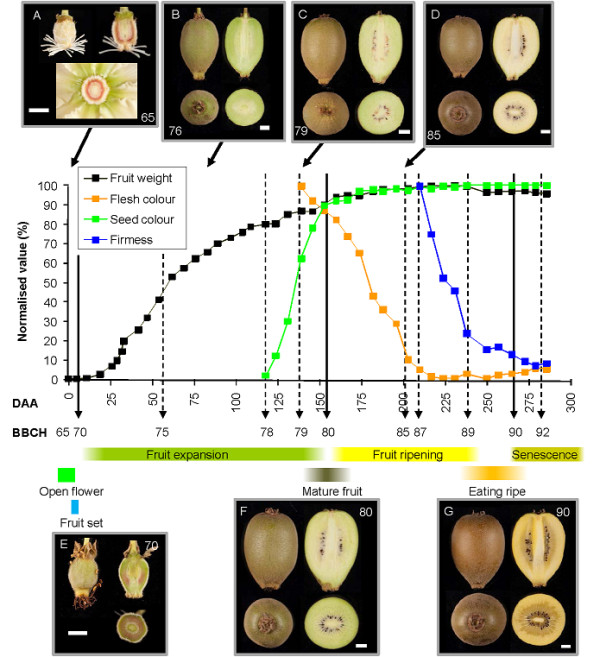
**Development of *Actinidia chinensis *'Hort16A'fruit from open flower (0 days after anthesis, DAA) to over ripe fruit (286 DAA) in Season 1**. The physiological changes have been scaled to 100 arbitrary units based on maximum values. Based on fresh weight (g), seed colour (% black), flesh colour (°h) and firmness (N), a BBCH scale has been aligned. A-E Photographs from different growth stages in Season 3 fruit with BBCH stages for each picture shown.

A detailed study of *A. chinensis *'Hort16A' during fruit development over three growing seasons (Seasons 1-3) was conducted from anthesis (0 Days After Anthesis, DAA) until fruit were senescing (300 DAA), and aligned to the BBCH scale (Figure 1, Additional File 1, 2). The principal stages of fruit growth that follow the fully opened flower (stage 65), are fruit set (stage 70) and mature fruit (stage 80). While many BBCH scales stop at stage 89 when the fruit is "eating ripe", for reasons given below, an additional principal fruit development stage has been added - fruit senescence (stage 90). This is consistent with the whole-plant BBCH scale, which allocates stage 90 and above for senescence, and stage 92 has been occasionally used for over-ripe fruit [30].

In 'Hort16A' fruit development, clear invariant descriptions for each of these principal growth stages are as follows:

**Principal stage 70 **Fruit set, petals abscised from the flowers and fruit growth starts

**Principal stage 80 **Mature fruit, seed within the fruit are over 95% black

**Principal stage 90 **Beginning of senescence, fruit start to produce autocatalytic ethylene.

In Season 1, these principal stages were attained at 10 DAA, 155 DAA and 270 DAA respectively (Figure 1E, F, G).

**Figure 2:**
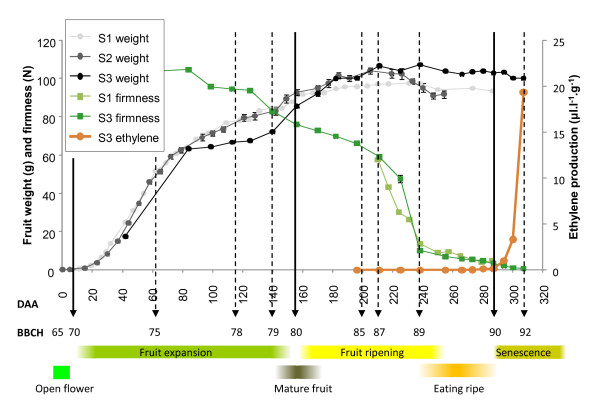
**Comparison of on-vine *Actinidia chinensis *'Hort16A' fruit growth over 3 seasons aligned to the BBCH scale**. Comparisons of fruit weight (g), firmness (N) and endogenous ethylene production (ul.l^-1^.g^-1^) are also presented. S1- Season 1, S2 - Season 2, S3 - Season 3.

For secondary growth stages within the BBCH scale, stages between 70 to 80 are described as a percentage of fruit growth, with stage 71 having 10% of final fruit weight, to stage 79 having 90% of final fruit weight. The pattern of fresh weight increase of 'Hort16A' Season 1 fruit was biphasic, following a sigmoidal growth curve (Figures 1, 2). After fertilisation, fruit growth increased exponentially, with fruit reaching stage 71 (10% of their final fresh weight) at 25 DAA, and stage 75 (50% final weight) at approximately 60 DAA. Following stage 75, the fruit entered a second, slower phase of growth, with stage 79 (90% of final weight) reached at 140 DAA. Further incremental increases in fruit weight were measured until stage 85 (200 DAA), after the seed had turned fully black and the flesh colour had changed. Thereafter fruit weight remained constant until stage 89 (237 DAA), when it decreased slightly as the fruit senesced (stage 90). At stage 78 (115 DAA), fruit growth had slowed considerably, and the seed had begun to change colour, firstly from white to brown, then finally to black (Figure 1C). Over 95% of seed had turned black at stage 80 (155 DAA; Figure 1F).

A similar sigmoidal pattern of growth was observed for fruit in Season 2 (Figure 2). However, Season 3 had an unusually hot dry period, which dramatically reduced fruit growth from stages 76 (60% of final fruit growth) to 78 (80 to 115 DAA). This slow growth was followed by a period of faster compensatory growth until approximately stage 83 (175 DAA) (Figure 2). This shows that unusual environmental conditions can change growth patterns, pushing growth beyond the point where seed are fully black. In these instances, the invariant developmental descriptor, seed greater than 95% black - stage 80, should be used.

For secondary stages between stages 80 to 90, the BBCH scale progresses from unripe fruit through to the start of fruit senescence. In many fruit species, change in flesh colour finishes at stage 85, at which point softening starts (stage 87), and the fruit softens to 'eating ripe' at stage 89. Eating ripe is a sensory descriptor that depends entirely on the consumer and therefore can vary considerably depending on personal taste. For a more reliable stage descriptor for 'Hort16A' fruit, a key point in development is the production of autocatalytic ethylene. This occurs at the end of fruit ripening and the beginning of fruit senescence (Figure 2). Autocatalytic ethylene production is a well defined and a readily measurable physiological event and has been assigned stage 90.

**Figure 3:**
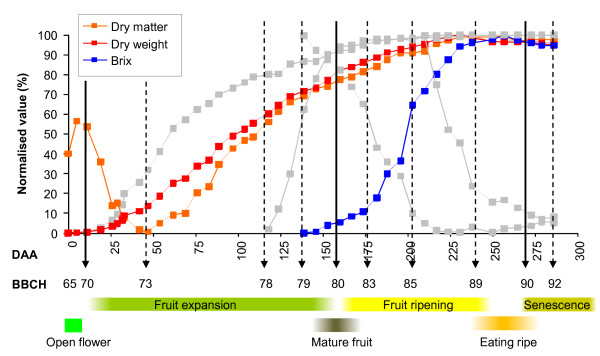
**Carbon accumulation in Actinidia chinensis 'Hort16A' fruit from Season 1**. Changes in dry weight (g), dry matter (%) and total soluble solids concentration (°Brix). Grey lines represent the fresh weight, seed colour, flesh colour and firmness detailed in Figure 1.

In 'Hort16A', the outer pericarp of immature fruit (< stage 79) has a green colour with a hue angle of approximately 115°. The colour starts to change at stage 79 (140 DAA), and progresses to a yellow colour (hue angle of approximately 100°h) by stage 85 (200 DAA) (Figure 1). At this time, the fruit is still firm (> 60 N). During stages 80-85, there is a significant increase in soluble sugars in the fruit; the inflection point of sugar increase is assigned stage 83 (175 DAA). The faster rate of sugar accumulation continues until the fruit is at stage 89 (237 DAA) (Figure 3).

In Season 3, fruit flesh firmness measurements were started as soon as the fruit were large enough to assess stage 75 (60 DAA). Between stage 75 and stage 85 (200 DAA), the fruit softened gradually from > 100 N to ~50 N (Figure 2). Following this point, and consistent with Season 1, the fruit underwent a typical kiwifruit softening pattern as found in 'Hayward' fruit. This softening pattern has been described as having four phases of softening [31]. Phase I is the initial drop in firmness starting at stage 87 (210 DAA), Phase II is the rapid drop in firmness (stage 88 - 225 DAA), and Phase III is the slow loss of firmness to ~10 N or less (finishing at stage 89 - 237 DAA). When the fruit produce autocatalytic ethylene, the cells produce aroma volatiles [32], and a further drop in firmness is observed (Phase IV softening), with fruit senescence starting - stage 90 (270-285 DAA) (Figures 1, 2). Stage 92 represents over-ripe senescent fruit, when the fruit are less than 4 N (> 300 DAA). In some less favourable growing conditions, the clear progression of secondary stages between stages 80 and 90 (flesh colour change, sugar accumulation, and softening) may not be observed, with flesh colour change occurring as late as stage 88 (J. Burdon, in preparation). In these instances, the more reliable markers for stage 83 (sugar inflection point) and stage 87 (start of rapid flesh softening) should be used (Table 1).

**Table 1:** Growth stages for Actinidia chinensis 'Hort16A'

Stage	BBCH description	BBCH *Actinidia chinensis* 'Hort16A'	Days after anthesis (DAA)
65	Fully open flowers	Fully open flower	0
**70**	**Fruit set**	**Fruit set; petals have abscised, fruit about to grow**	**10**
71	10% fruit growth	Fruit reached 10% final weight	25
72		Fruit reached 20% final weight	
73		Fruit reached 30% final weight; minimum dry matter	45
74		Fruit reached 40% final weight	
75	50% fruit growth	Fruit reached 50% final weight; growth slowing	60
76		Fruit reached 60% final weight	
77		Fruit reached 70% final weight; maximum acids	100
78		Fruit reached 80% final weight; seeds start to change colour	115
79	Fruit finished growth	Fruit reached 90% final weight	140
**80**	**Fruit mature**	**Mature fruit; seeds 95% black; outer pericarp starts to change colour**	**155**
83		Start of rapid increase in soluble sugars	175
84		Maximum starch	190
85	Colour change finished	Outer pericarp turned yellow (100° hue angle)	200
87	Softening starts	Start of flesh softening	210
88		Rapid flesh softening	225
89	Eating ripe	Fruit 10 N firmness softening slows	237
**90**	**Plant senescence**	**Production of autocatalytic ethylene**	**270-285**
92		Fruit less than 4 N, senescence	> 300

### Change in fruit morphology over development

**Figure 4:**
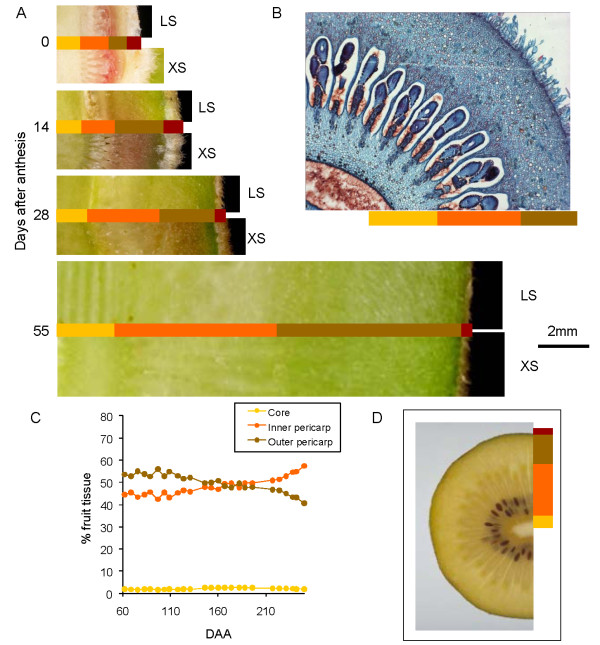
**Tissue growth in developing *Actinidia chinensis *'Hort16A' fruit**. Core tissue (yellow), inner pericarp (orange), outer pericarp (khaki), epidermis (maroon). A) Early growth stages, kiwifruit carpels and early fruit sections to stage 74 (55 days after anthesis, DAA). XS - cross section, LS - Longitudinal section. B) Structure of a cross section of an unfertilised kiwifruit carpel, showing the distribution of the different tissue types. At the centre of the fruit is the invagination of the centre of the carpel (stained brown). C) A summary of the growth from stages 75 (60 DAA) to stage 89 (238 DAA), and D) a cross section area of a fruit starting senescence (stage 90).

'Hort16A' fruit develop from a multicarpellate ovary, with each carpel containing a number of ovules. The fruit has four distinct tissue types: a central core, an inner pericarp (IP) containing locules and seed, a dense outer pericarp (OP), and the skin (Figure 4A-D). At anthesis (fully open flower - stage 65), the ovules are attached to the core, which is surrounded by pericarp tissue that is also attached to the core by filaments a few cells thick (Figure 4B). At this point, the skin is covered with a thick hair (Figure 1 and 4A). By stage 70, the OP tissue has expanded to three times its original width in the flower, while the locular cavities, which develop into the IP, have not expanded at this stage (Figure 4A). By stage 71, the developing IP has expanded rapidly surrounding the seed (Figure 4A). By stage 75, the IP makes up approximately 40% of the fruit cross sectional area of the fruit, after which the IP continues to expand more rapidly than the OP throughout the rest of fruit growth. As the fruit ripens, the inner pericarp zone extended towards the OP, resulting in the OP being a band of tissue only a few millimetres deep adjacent to the skin, once the fruit reaches stage 90 (Figure 4C).

### Carbon accumulation during growth

While the fresh weight accumulation of 'Hort16A' fruit shows a sigmoidal growth pattern with slowing growth after stage 75, the dry weight of fruit increases linearly between stage 71 (28 DAA) and stage 79 (140 DAA) (Figure 3). After this point, the fruit continues to increase in dry weight, reaching a maximum dry weight at stage 89 (237 DAA), considerably later than the fresh weight maximum observed at stage 85 (200 DAA) (Figure 3). This difference suggests a complex relationship between carbon and water accumulation during development which may be visualised by comparing fresh and dry weight of fruit as dry matter percentages (Figure 3). The dry matter content of the fruit is high at fruit set (stage 70), and then rapidly drops during the first period of rapid growth, reaching a minimum at 45 DAA. This is when the fruit has reached 30% of the final fresh weight - stage 73 (Table 1). The dry matter then increases more rapidly than the fresh weight, until stage 89, at which point no further increase in dry matter is observed (Figure 3).

**Figure 5:**
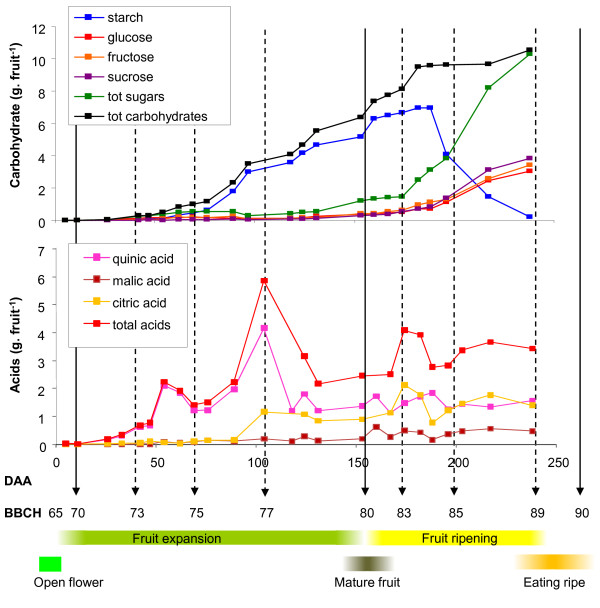
**Non-structural carbohydrate (A) and organic acid (B) composition of *Actinidia chinensis *'Hort16A' fruit during development from open flower (stage 65) to eating ripeness (stage 89) during Season 1**.

From stage 73 onwards, starch begins to accumulate in the fruit until a starch maximum at 190 DAA (stage 84) (Figure 5A). Thereafter, starch is rapidly broken down and metabolised to similar concentrations of sucrose, glucose and fructose (Figure 5A). The amount of soluble sugars in 'Hort16A' fruit continues to increase until the fruit reaches eating ripeness. When the sugar content in each tissue was assessed, the OP and IP both had similar composition until the fruit began to ripen. However, during ripening the starch was more rapidly converted into sugars in the OP than in the IP (Additional File 3). Interestingly, the inflection point of soluble sugar increase occurred before any marked decrease in starch was observed (at 175 DAA -stage 83).

During the early stage of rapid fruit growth (stages 71-77 (70% of final fruit weight)) accumulation of acids, particularly quinic acid was observed (Figure 5B). This increase has been previously reported in other *Actinidia *species [33] and shown to be part of the osmotic potential in the fruit, aiding fruit growth. After this point, there was a rapid decrease in total acids to stage 79, after which a steady state of acids was observed in the fruit. From stage 76, there was an increase in citric acid, after which this remained constant through the rest of fruit growth. Malic acid was a minor fruit acid accumulated during development (Figure 5B).

### Fruit ethylene responses

**Figure 6:**
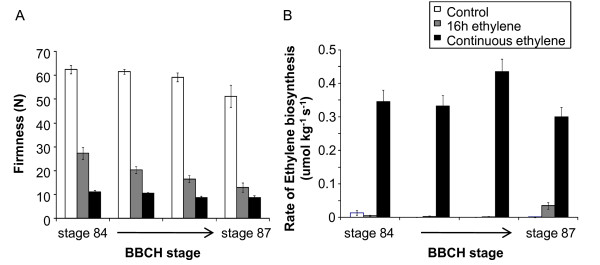
**Softening (A) and ethylene production (B) of *Actinidia chinensis *'Hort16A' fruit treated with 100 ul**.l^-1 ^ethylene at different stages of development. Fruit were treated with either a 16-hour ethylene treatment (grey), or a continuous ethylene treatment for two days (black), and compared with control untreated fruit (white).

In 'Hort16A' fruit, many of the physiological changes associated with fruit ripening (starch conversion, colour change, and flesh softening) occur in the absence of any marked increase in ethylene production (Figure 2). Indeed, when the fruit is left on the vine, autocatalytic ethylene was only detected as the fruit progresses into senescence - stage 90. While there is no measurable increase in ethylene production by the fruit during ripening, the capacity of the fruit to respond to exogenous ethylene develops before this time. The application of exogenous ethylene to detached fruit from stage 84 can rapidly accelerate fruit softening to eating ripeness (stage 89; Figure 6A). Subsequent weekly fruit harvests through to stage 87 showed little softening in the fruit left on the vine, but the sensitivity to ethylene increased during this time (Figure 6A). In early harvested fruit, the degree of softening was dependent on the duration of ethylene application. Continuous ethylene (100 ul.l^-1^) supply resulted in greater softening than a discrete application of ethylene (16 h, 100 ul.l^-1^). The difference in firmness after 2 days between fruit treated with either discrete or continuously applied ethylene was reduced from about 18 N in fruit at stage 84 to < 5 N in fruit at stage 85. The rapid softening observed in the ethylene treated fruit did not necessarily coincide with an increase in endogenous autocatalytic ethylene production (Figure 6B), as fruit treated for 16 hours (50 ul.l^-1^) did not produce autocatalytic ethylene, while the continuously ethylene-treated fruit did.

**Figure 7:**
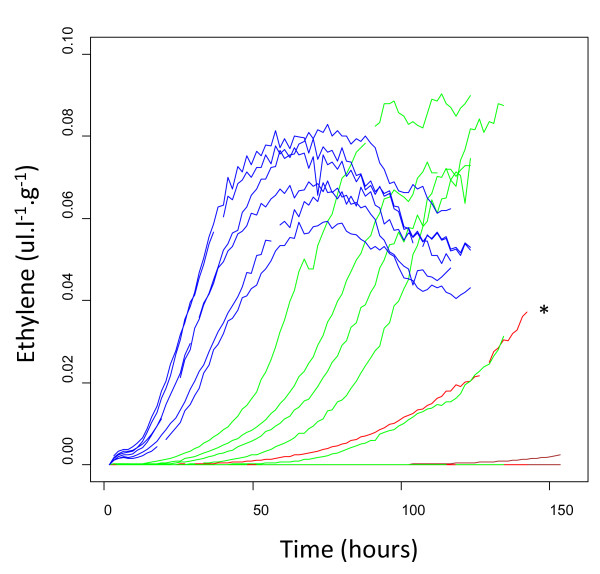
**Ethylene produced by *Actinidia chinensis *'Hort16A' fruit after treatment with 50 ul.l^-1 ^exogenous ethylene for different periods**. Three treated fruit were placed in jars with continuous air flow (25 ml. min^-1^) and monitored for ethylene production. Black lines were not treated with ethylene, red lines 12-h treatment, green lines 24-h treatment, blue lines 36-h treatment, * marks indicates the 12-hour treated fruit containing an infected fruit.

To establish the threshold of ethylene needed to induce autocatalytic ethylene production, fruit at stage 88 were treated for different periods of 50 ul.l^-1 ^ethylene (12, 24, 36 hours) and continuously monitored for ethylene production over the subsequent seven days. None of the control (untreated) fruit produced autocatalytic ethylene, and only 1 of the 12-hour-treated fruit produced autocatalytic ethylene (this one jar was later discovered to have a disease-infected fruit in it) (Figure 7). However, 5/6 of fruit treated with ethylene for 24 hours produced autocatalytic ethylene between 24 hours to 7 days following removal from ethylene treatment. All fruit treated with ethylene for 36 hours produced ethylene within 24 hours of treatment. The increase in ethylene correlated to an increase in respiration rate (Additional File 4). Following the 7-day period of ethylene measurements, flesh firmness was assessed. Fruit from jars with no detectable ethylene remained at around 8 N, which is consistent with stage 89, whereas the fruit from jars producing autocatalytic ethylene had progressed to stage 92 and beyond, with a firmness of less than 4 N.

### Change in gene expression over fruit development

**Figure 8:**
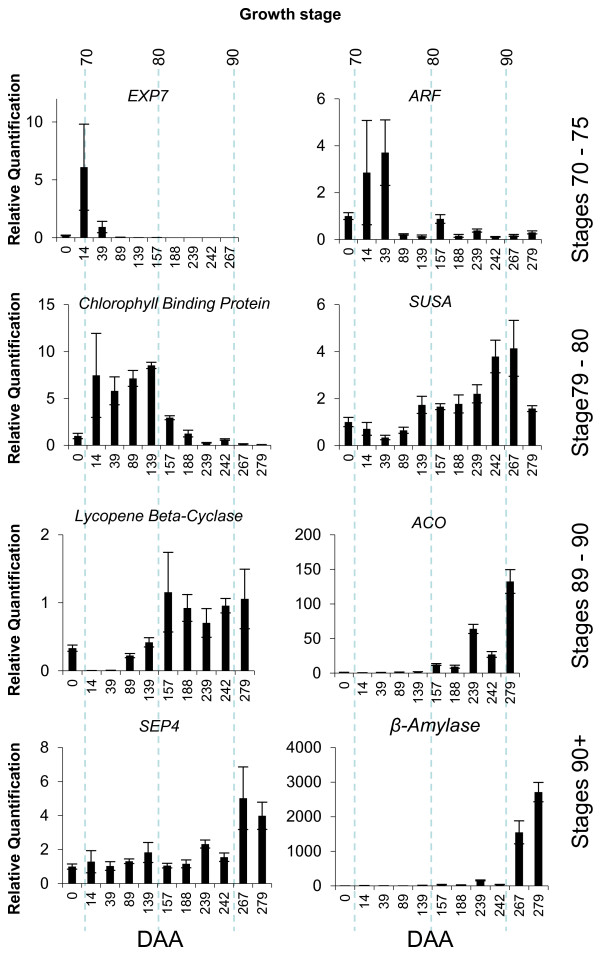
**Eight genes giving different expression patterns over *Actinidia chinensis *'Hort16A' fruit development**. *EXPANSIN 7 *(*EXP7*) [FG499166], *AUXIN RESPONSE FACTOR *(*ARF*) [FG482624], *CHLOROPHYLL A/B BINDING PROTEIN *[FG527942], *SUCROSE SYNTHASE A *(*SUSA*) [FG439911], *LYCOPENE β-CYCLASE *[FG486019], *ACC OXIDASE *[FG460245], *SEPALLATA 4 *(*SEP4*) [FG460077], *β-AMYLASE *[FG525163]. Expression patterns are given relative to EIFα.

It is anticipated that future work that uses this scale would include molecular studies. To cater for this, we aimed to extend the physiological scale to include genes with differential expression over fruit development, which may be used as marker genes for the different stages. Twenty genes were selected for expression analysis, based on literature searches and the presence of sequences in individual fruit tissue libraries in the kiwifruit EST collection. Genes were selected with functions that may explain some of the phenotypic changes observed [13]. Fourteen of these genes showed a single amplification product and demonstrated differences in expression during fruit development. These included a *POLYGALACTURONASE *(*PG*) [34], *ACC OXIDASE *[32], two *EXPANSIN *(*EXP*) genes, an *AUXIN RESPONSE FACTOR *(*ARF*) and *CHLOROPHYLL A-B BINDING PROTEIN *(*CAB*), a *LYCOPENE CYCLASE *[17], a *SEED MATURATION PROTEIN*, a *SEPALLATA 4 *(*SEP4*) like gene [35] and four genes associated with starch metabolism, including a *β-AMYLASE *and *SUCROSE SYNTHASE A *(*SUSA*) [36] (Additional File 5). Expression of the eight genes that best marked different stages of 'Hort16A' fruit development is shown in Figure 8. Expression of the remaining genes overlapped these patterns (Additional File 6). During the early stages of development, expression of *EXP7 *peaked at stage 71 and the *ARF *was highly expressed during stages 71-75. The expression of a *CAB *increased during stages 71-80. Expression of the *SUSA *gene increased in fruit at stage 8 and remained high until fruit senesced (91). The starch plastid protein showed increased expression in fruit from stage 85 through to eating ripeness (stage 89), while the *SEP4 *and *β-AMYLASE *genes were highly expressed in senescing fruit after stage 89 (Figure 8).

## Discussion

**Figure 9:**
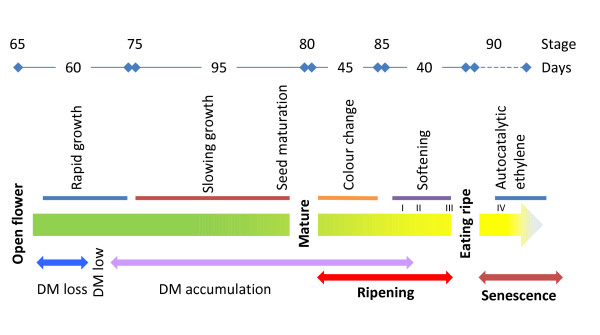
**Schematic diagram of kiwifruit growth and development**. Stages are marked above with time intervals (days) between each stage. DM is percentage dry matter of the fruit, and the ripening and senescence phases shown. The four phases of softening are marked in Roman numerals.

Here we have described in detail the physiological changes that occur during on-vine development of *Actinidia chinensis *'Hort16A' fruit, from anthesis to senescence, in relationship to the BBCH scale (summarised in Figure 9). The BBCH scale is a whole-plant scale, designed by crop physiologists to compare growth and development from seed to senescence across plant species, with fruit being only one component of the scale. This results in some studies providing only a coarse description of fruit growth stages, which are hard to apply to a given experiment. With the detailed descriptions of fruit growth given here (Table 1), a framework is provided for fruit development studies in other cultivars, and other *Actinidia *species. This scale differs from the scale proposed in a whole-plant study for a second kiwifruit species, *A. deliciosa *'Hayward' by [28]. Firstly, the principal stage 80 is assigned to fully black seed (previously assigned to stage 85 [28]), and secondly, stage 90 is assigned to fruit that are beginning to produce autocatalytic ethylene, which was not measured in [28]. The invariant principal stages detailed here are likely to be conserved across *Actinidia*, and secondary stages between 70 and 80 based on final fruit size can easily be translated. The secondary stages between 80 and 90 are likely to be more species- and cultivar-specific, especially with the range of flesh colour in ripe fruit observed across different *Actinidia *species and cultivars [13,18]. The descriptor for stage 90 appears to be conserved, as previous studies in other *Actinidia *species have reported a ripening progression in the absence of autocatalytic ethylene [11,21,37].

Like many perennial fruit, such as apple [38], grape [26], and citrus [1], 'Hort16A' kiwifruit have a long fruit development period. Fruit growth spans much of the annual growing season, beginning at anthesis in spring, followed by rapid summer fruit growth, ripening in autumn and finally senescence and abscission of fruit from the vine in winter [7,39]. In contrast, fruit growth and development in tomato occurs over only 1.5 to 2 months, compressing the events of fruit development [3,40,41]. Despite the difference in time to develop, tomato and 'Hort16A' fruit have many similarities; they are true fruit (berries) that are ovary-derived, they have similar tissue zones, (outer pericarp, locular inner pericarp and core tissue) and during maturity and ripening the fruit flesh changes colour and the flesh softens. In this study, 'Hort16A' fruit growth followed a sigmoidal pattern, in agreement with previous studies [15]. The closely related *A. deliciosa *fruit growth has also been described as having a double [7] or triple [11] sigmoidal growth pattern. In these studies, the additional inflections are likely to be environmentally derived rather than genetically programmed, as shown by the slower period of growth in 'Hort16A' during drought in Season 3. Biphasic growth is also typical of tomato fruit from a range of different genotypes [40]. In this study, we have also documented that fruit growth continues during the early stages of fruit ripening, and fruit appear to shrink once they reach eating ripeness and begin to senesce. In both tomato and *A. deliciosa*, the first stage of growth is dominated by a period of cell division, seed formation and early embryo development [3,7], which is likely to be true for 'Hort16A' in the initial period of development (stages 70-75). This period was characterised by rapid changes in the size and development of first the outer and then the inner pericarp tissue, development of seed, as well as expression of an *EXP7 *and an *ARF *gene. Another feature of this period of rapid growth and development was the greater influx of water compared with carbon into fruit [15] and accumulation of quinic acid [42]. The fundamental change from the initial rapid period of growth to a longer period of slowing growth is defined as stage 75. In both tomato and 'Hayward' kiwifruit, this change in growth rate indicates the change from predominantly cell division to a period of cell expansion [3,7].

Compared with many other fruit, the ripening response in *A. chinensis *is unusual. The pattern of ethylene production of 'Hort16A' fruit is similar to that of 'Hayward' fruit. 'Hayward' has been classified as a climacteric fruit [43], despite the fruit ripening in the absence of a marked increase in ethylene production [11]. In 'Hayward', fruit typically do not produce autocatalytic ethylene until below a firmness of 10 N [21], unless damaged in some way, either physically, physiologically or by disease [44,45]. 'Hort16A' also ripens in the absence of autocatalytic ethylene production. This contrasts with tomato, where ethylene production increases rapidly at the beginning of fruit ripening. Autocatalytic ethylene is only produced by 'Hort16A' fruit, after stage 89, and therefore it may therefore be more appropriate to link ethylene production with fruit senescence rather than ripening. Because of this, we have included stage 90 for senescence in the proposed BBCH scale for 'Hort16A' (Figure 9). It is important to note, however, that this senescence phase appears to be linked to a tomato ripening response as the *RIPENING INHIBITOR *gene [46] is similar to the Arabidopsis *SEP4 *gene. The closest kiwifruit homologue of this gene *AcSEP4 *[35] is upregulated at the point of climacteric ethylene production. This also corresponds to other ripening-associated gene expression; for example, up-regulation of a *β-AMYLASE *gene [32] was observed during this phase (Figure 8).

While kiwifruit ripening is independent of autocatalytic ethylene production, it appears that endogenous ethylene may play a role in softening. Studies with the inhibitor of ethylene action 1-Methylcyclopropene (1-MCP) have shown that rapid softening (stage 88) can be slowed by blocking ethylene action [47,48]. In addition, kiwifruit are particularly sensitive to exogenously applied ethylene and respond by softening rapidly [49]. This suggests that basal amounts of ethylene may contribute to the ripening process, while not actually inducing an autocatalytic response. It also suggests that autocatalytic ethylene production has a lower sensitivity to ethylene than fruit softening, which is consistent with the sensitivity/dependency model proposed for apple fruit ripening by.

## Conclusions

This study has detailed the developmental changes that occur during fruit development in 'Hort16A' fruit. By aligning this to a BBCH scale, we provide a framework for further physiological and molecular studies to be compared, independently of the number of days after anthesis, or environmental effects in any given year.

## Methods

### Plant material and harvest dates

Experiments were carried out using fruit from *Actinidia chinensis *(Planch.) var. *chinensis *'Hort16A' vines grown at Plant & Food Research, Kerikeri Research Centre (35°14' S 173°55' E) during the 2004/2005 (Season 1), 2005/2006 (Season 2) and 2009/2010 (Season 3) growing seasons. Season 1 flowers open at 50% anthesis (21 October 2004) were tagged for subsequent sampling, with two fruit from each of ten vines sampled at weekly intervals and assessed as below. Season 2 repeated the design in Season 1, with weekly measurements started at 50% anthesis (16 October 2005). Fruit from Season 3 were sampled at 2-weekly intervals from 50% anthesis (10 October 2009) photographed, and tested for firmness and ethylene production (Figures 1, 2 and Additional file 1, 2). For postharvest treatments, in Season 3 bulk harvests of fruit from 10 vines were assessed for response to ethylene, starting at stage 84.

### Fruit assessment methods

Following sampling, individual fruit were weighed. Fruit firmness was determined either using an Effigi penetrometer (Alphonsine, Italy), or a Fruit Texture Analyser (Guss, South Africa) with a 7.9-mm probe at 20 mm.s^-1 ^[50] following removal of a 1-mm thick slice of skin and outer pericarp at two locations at 90° at the fruit equator. A 2-mm equatorial slice was taken to determine the proportions of inner and outer pericarp tissue, by measuring the maximum and minimum diameter of each tissue with digital callipers (Mitutoyo, Japan) and the percentage of fully coloured black seed in fruit were recorded. This slice was then weighed and dried at 65°C for 24 hours to determine fruit dry weight and percentage fruit dry matter. Dry matter was calculated as dry weight/fresh weight * 100. Flesh colour was determined using a CR 300 Chroma meter (Minolta, Japan), using a C65 light source and the LCH colour system, at two positions on each fruit at 90° at the equator following removal of skin and flesh to a depth of about 2 mm [16]. Soluble solids were determined for juice taken from both ends of the fruit using a refractometer (Atago, Japan) [51].

### Ethylene treatments

Bulk samples from Season 3 were collected at weekly intervals from stage 84 to stage 87. For each sampling time, two thirds were treated with 100 ul.l^-1 ^ethylene in 340-L containers with circulating air. A third were removed after a 16-hour treatment and a third were left for 2 days; the remaining fruit were left in an ethylene-free environment. Before assessment, ethylene was vented for 2 hours and fruit were measured for firmness and endogenous ethylene production. Ethylene production was measured by sealing individual fruit in 536-ml container at 20°C. 1 ml of headspace was assessed by gas chromatograph fitted with a flame ionisation detector as described in [32], ethylene production was calculated as a rate per gram of fruit.

For continuous ethylene monitoring (Figure 7), ethylene-treated fruit were divided into 6 batches of fruit per treatment (3 fruit per batch). Fruit were exposed to 50 ul.l^-1 ^ethylene for 12, 24 and 36 hours as above and placed into individual (1l) jars with continuous air flowing (28 ml.min^-1^) over them. Every 2 hours, a sample of the air was assessed for ethylene using a GC 2014 (Shimadzu, Japan).

### Non-structural carbohydrates and organic acid composition

Season 1 fruit were sampled taking a longitudinal slice of fruit tissue (representing all tissue types) from each of a minimum of four fruit, snap frozen in liquid nitrogen and stored at -80°C for later analysis of non-structural carbohydrate (starch and soluble sugars) and organic acids. Starch was analysed as previously described by [52], soluble sugars (glucose, fructose, sucrose, *myo*-inositol and galactose) were measured as described by [53], whereas organic acids (citric, quinic and malic acids) were determined following the protocol described by [54].

### Gene expression analysis

Season 1 fruit were also sampled for gene expression analysis. For early season samples, 0 - 60 DAA, at least 10 independent fruit were harvested for RNA extraction. After 60 DAA, an equatorial slice from each of 10 fruit was taken for analysis. Each sample was snap frozen in liquid nitrogen and stored at -80°C until extracted. RNA was extracted as described by [55], and cleaned using an RNA cleanup kit (Qiagen) according to the manufacturer's protocol. Single-stranded cDNA was synthesised using the SuperScript III (Invitrogen) kit according to the manufacturer's protocols. Quantitative PCR was used as described in [32], using the PP2a and EF1α gene as a house keeping genes. In both cases the expression patterns of the genes were similar. Expression patterns found in figure are compared to Actin. Primers for qPCR are detailed in Additional File 5.

## List of Abbreviations

BBCH: Biologische Bundesantalt, Bundessortenamt und Chemische Industrie; DAA: Days After Anthesis; EST- Expressed Sequence Tag; IP: Inner Pericarp; OP: Outer Pericarp; qPCR: quantitative Polymerase Chain Reaction.

## Authors' contributions

ACR conceived and designed experiment, undertook orchard data analysis, and helped draft the manuscript, HLB undertook the carbohydrate and acid measurements and data analysis, PAM undertook the postharvest physiology and molecular sample processing and expression analysis, KG undertook postharvest physiology and molecular sample processing, ZL helped with the gene expression analysis, RGA undertook data analysis and helped draft the paper, KMD participated in data analysis and helped draft the paper, JMB helped with postharvest analysis and interpretation and helped draft the paper, RJS conceived and designed the experiment, analysed the data, coordinated the paper, and helped draft the manuscript. All authors have read and approved the manuscript

## Supplementary Material

Additional file 1 **Fruit from *Actinidia chinensis *'Hort16A' recorded every two weeks through development, longitudinal section**. Click here for file

Additional file 2 **Fruit from *Actinidia chinensis *'Hort16A' recorded every two weeks through development, cross section**. Click here for file

Additional file 3 **Sugar composition of different tissue types through fruit development**. Click here for file

Additional file 4 **Ethylene and CO_2 _production in fruit producing autocatalytic ethylene**. Click here for file

Additional file 5 **Details of qPCR primers and GenBank accession of the genes**. Click here for file

Additional file 6 **Expression analysis of other genes changing over fruit development**. Click here for file
