# Supplementary figures and images for: Fruit development of the diploid kiwifruit, Actinidia chinensis 'Hort16A'
Source: BMC Plant Biol. 2011 Dec 28;11:182. doi: 10.1186/1471-2229-11-182 (PMC3261216; doi:10.1186/1471-2229-11-182)

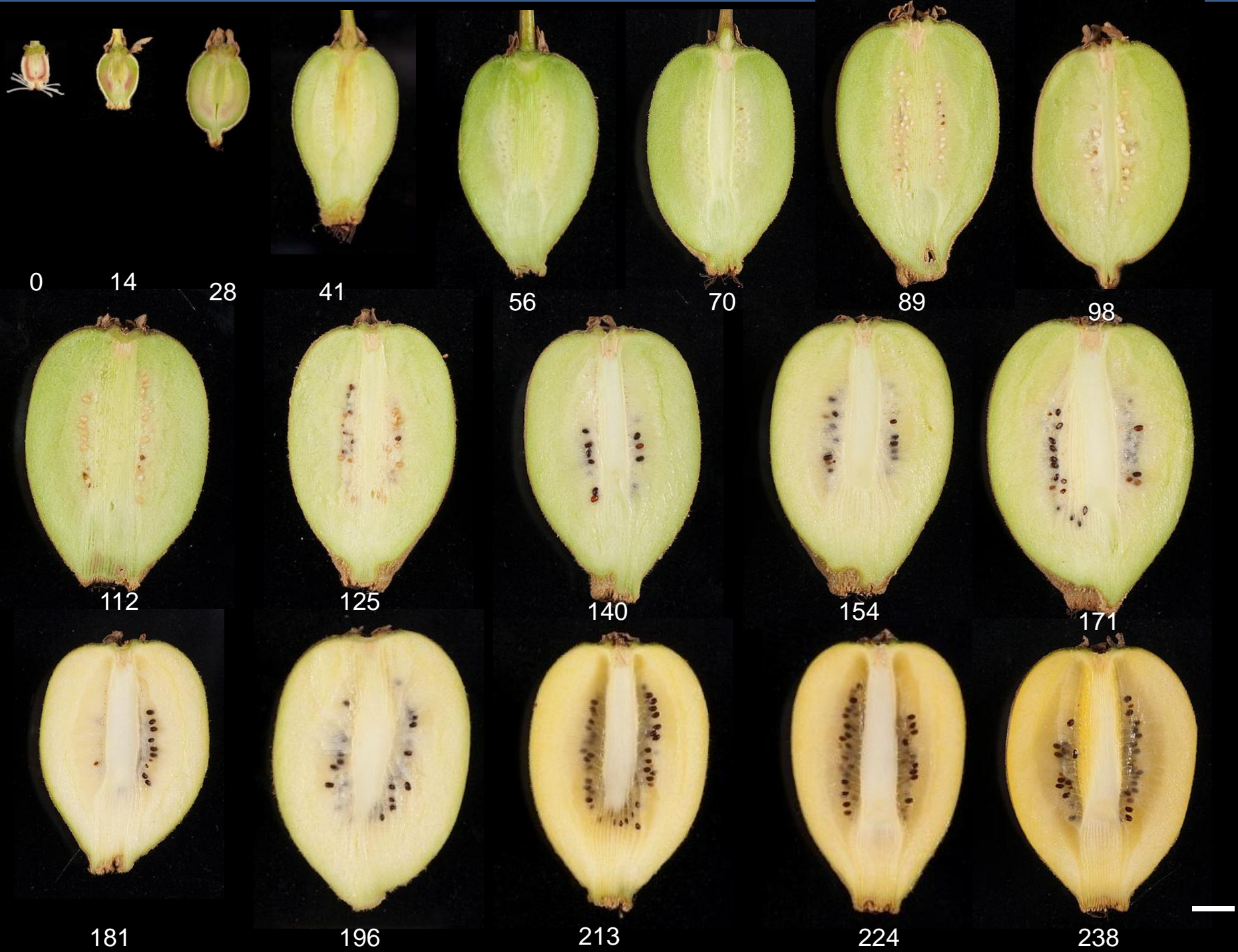

Supplement: Additional file 1 — Fruit from Actinidia chinensis 'Hort16A' recorded every two weeks through development, longitudinal section. [file 1471-2229-11-182-S1.PDF]

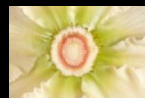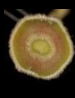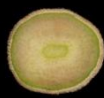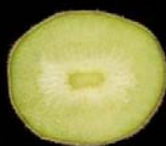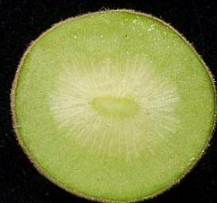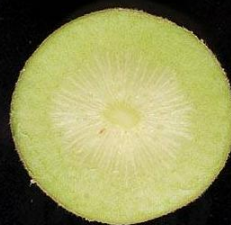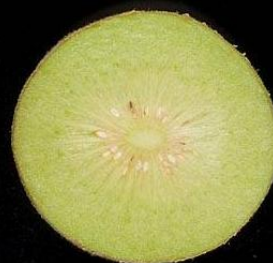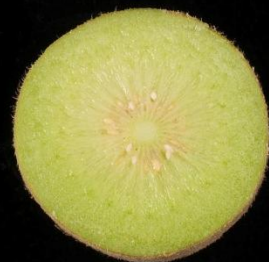

0

14

28

41

56

70

89

98

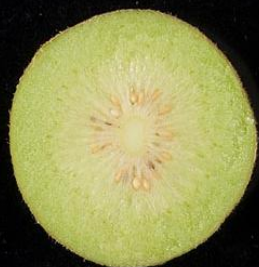

112

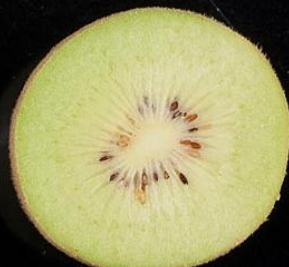

125

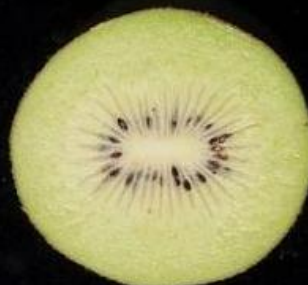

140

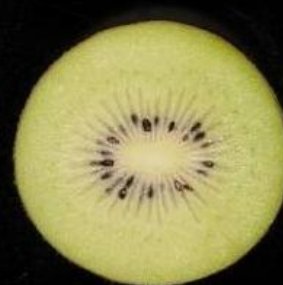

154

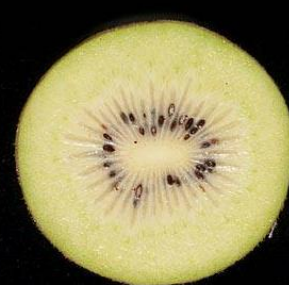

171

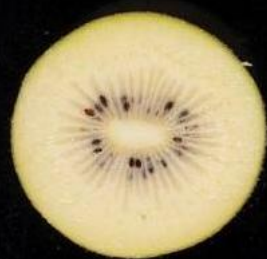

181

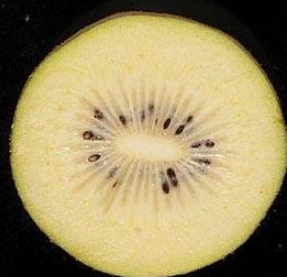

196

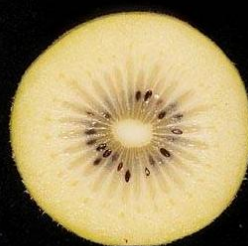

213

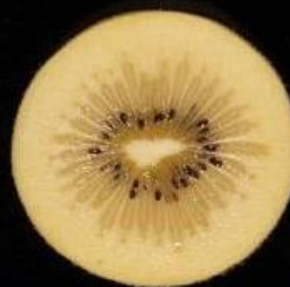

224

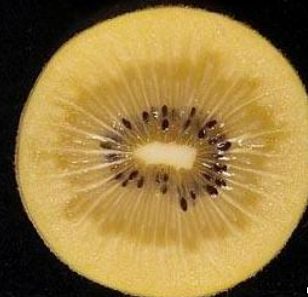

238

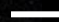

Supplement: Additional file 2 — Fruit from Actinidia chinensis 'Hort16A' recorded every two weeks through development, cross section. [file 1471-2229-11-182-S2.PDF]

## Slide 1
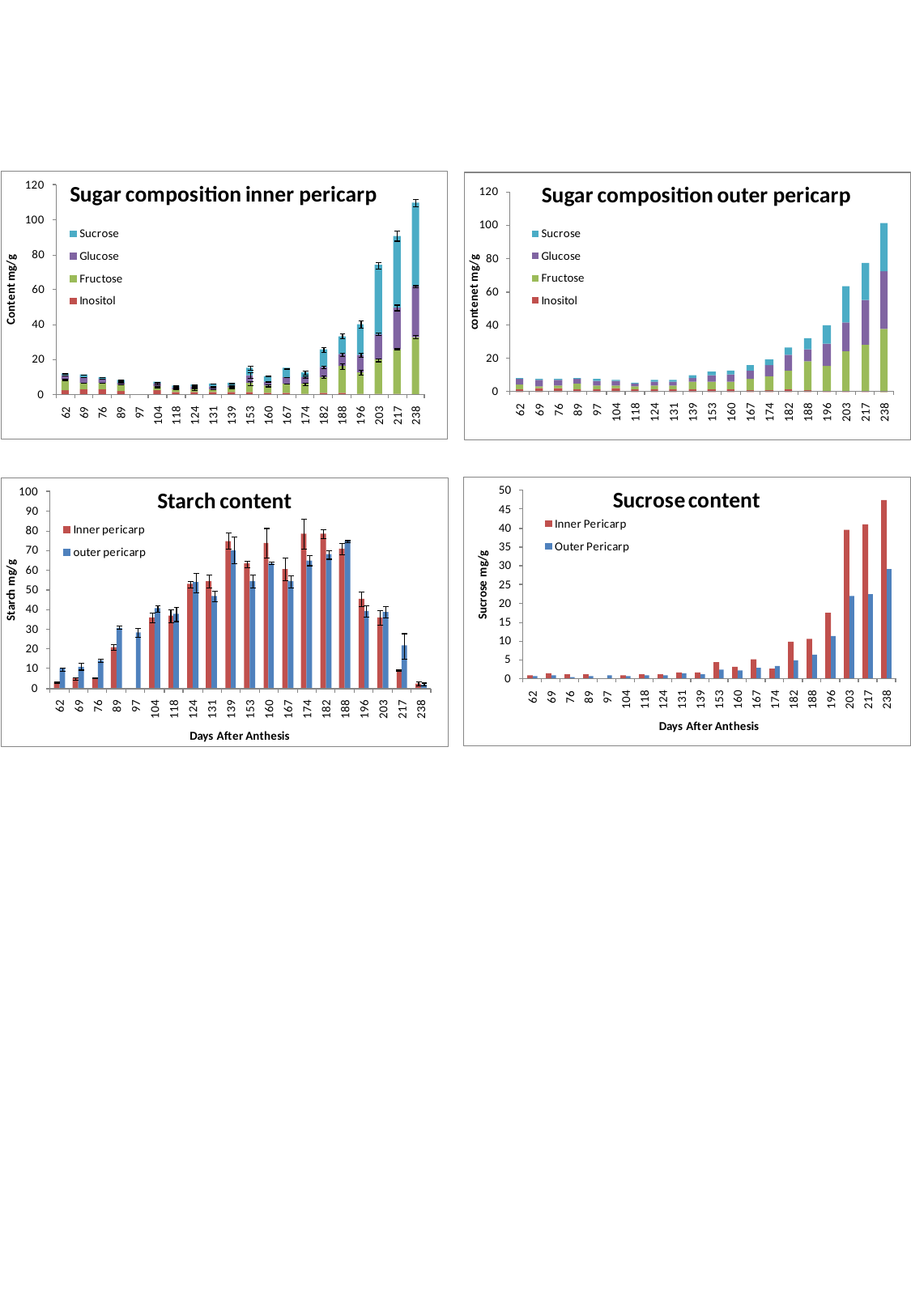

Supplement: Additional file 3 — Sugar composition of different tissue types through fruit development. [file 1471-2229-11-182-S3.PPT]

## Slide 1
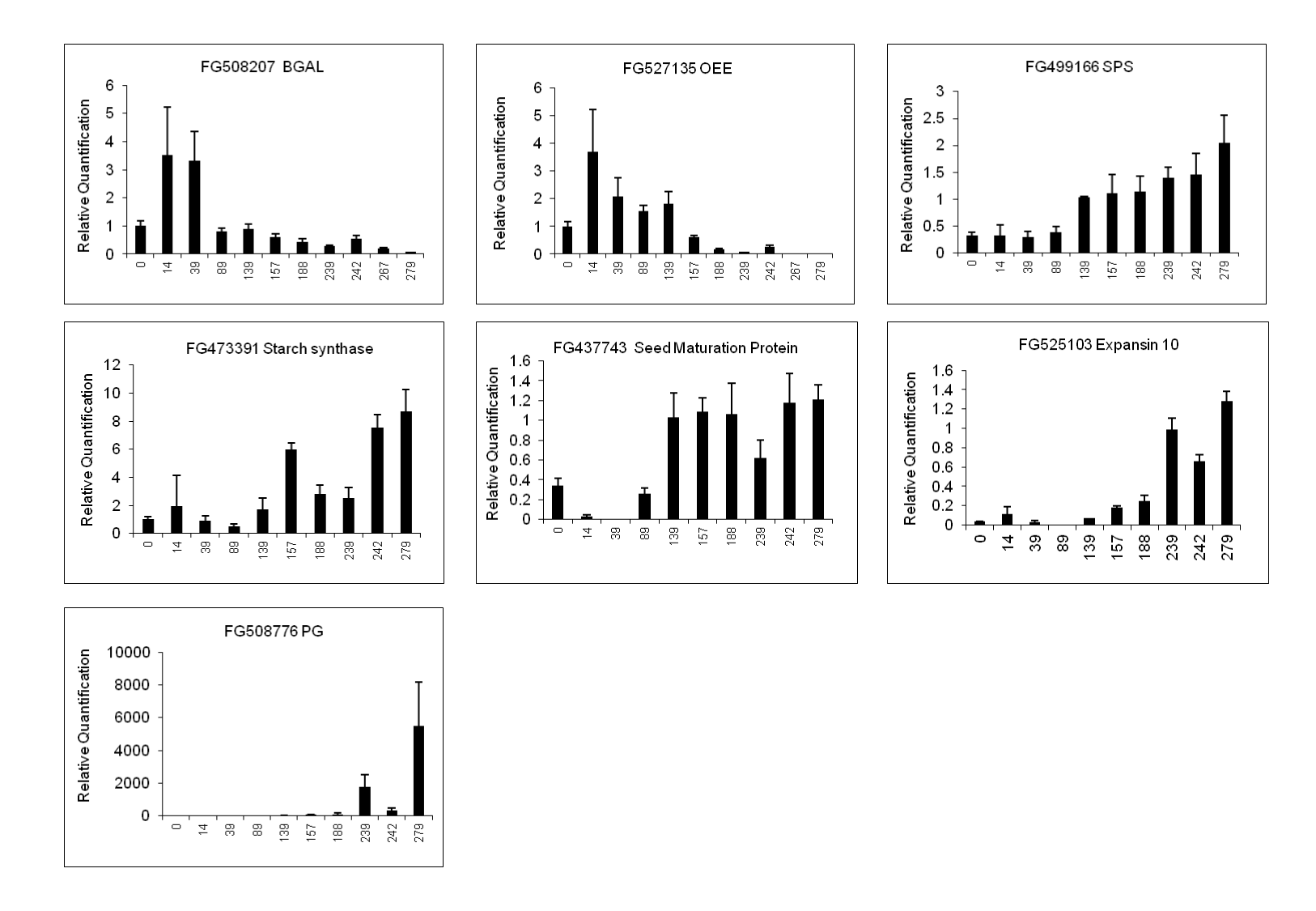

Supplement: Additional file 6 — Expression analysis of other genes changing over fruit development. [file 1471-2229-11-182-S6.PPT]
