# Supplementary material for: Fruit development of the diploid kiwifruit, Actinidia chinensis 'Hort16A'
Source: BMC Plant Biol. 2011 Dec 28;11:182. doi: 10.1186/1471-2229-11-182 (PMC3261216; doi:10.1186/1471-2229-11-182)
Supplement: Additional file 4 — Ethylene and CO2 production in fruit producing autocatalytic ethylene. [file 1471-2229-11-182-S4.PPT]

## Slide 1
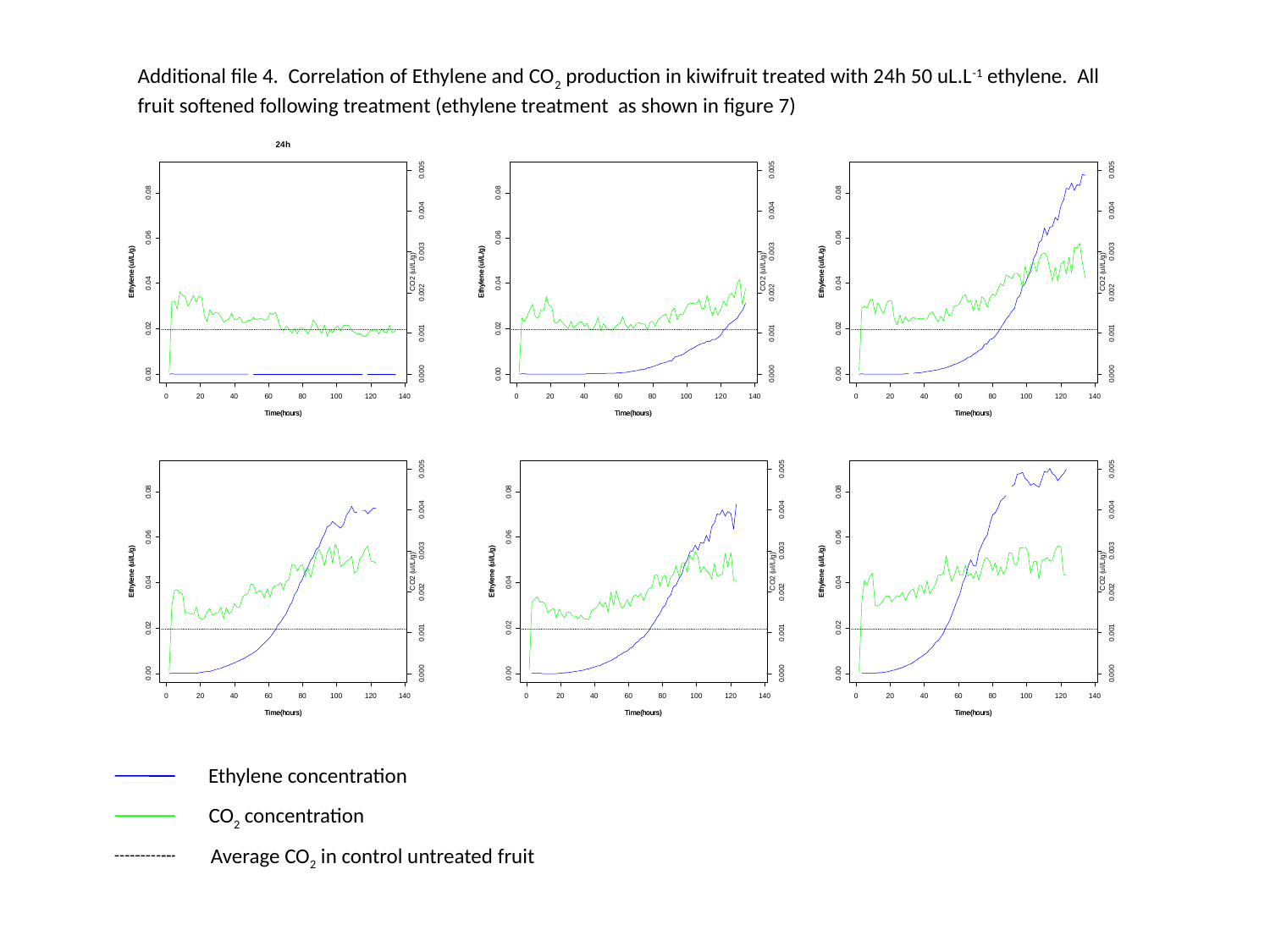

Additional file 4. Correlation of Ethylene and CO2 production in kiwifruit treated with 24h 50 uL.L-1 ethylene. All fruit softened following treatment (ethylene treatment as shown in figure 7)
Ethylene concentration
CO2 concentration
Average CO2 in control untreated fruit
