# Supplementary material for: Fruit development of the diploid kiwifruit, Actinidia chinensis 'Hort16A'
Source: BMC Plant Biol. 2011 Dec 28;11:182. doi: 10.1186/1471-2229-11-182 (PMC3261216; doi:10.1186/1471-2229-11-182)
Supplement: Additional file 5 — Details of qPCR primers and GenBank accession of the genes. [file 1471-2229-11-182-S5.PPT]

## Slide 1
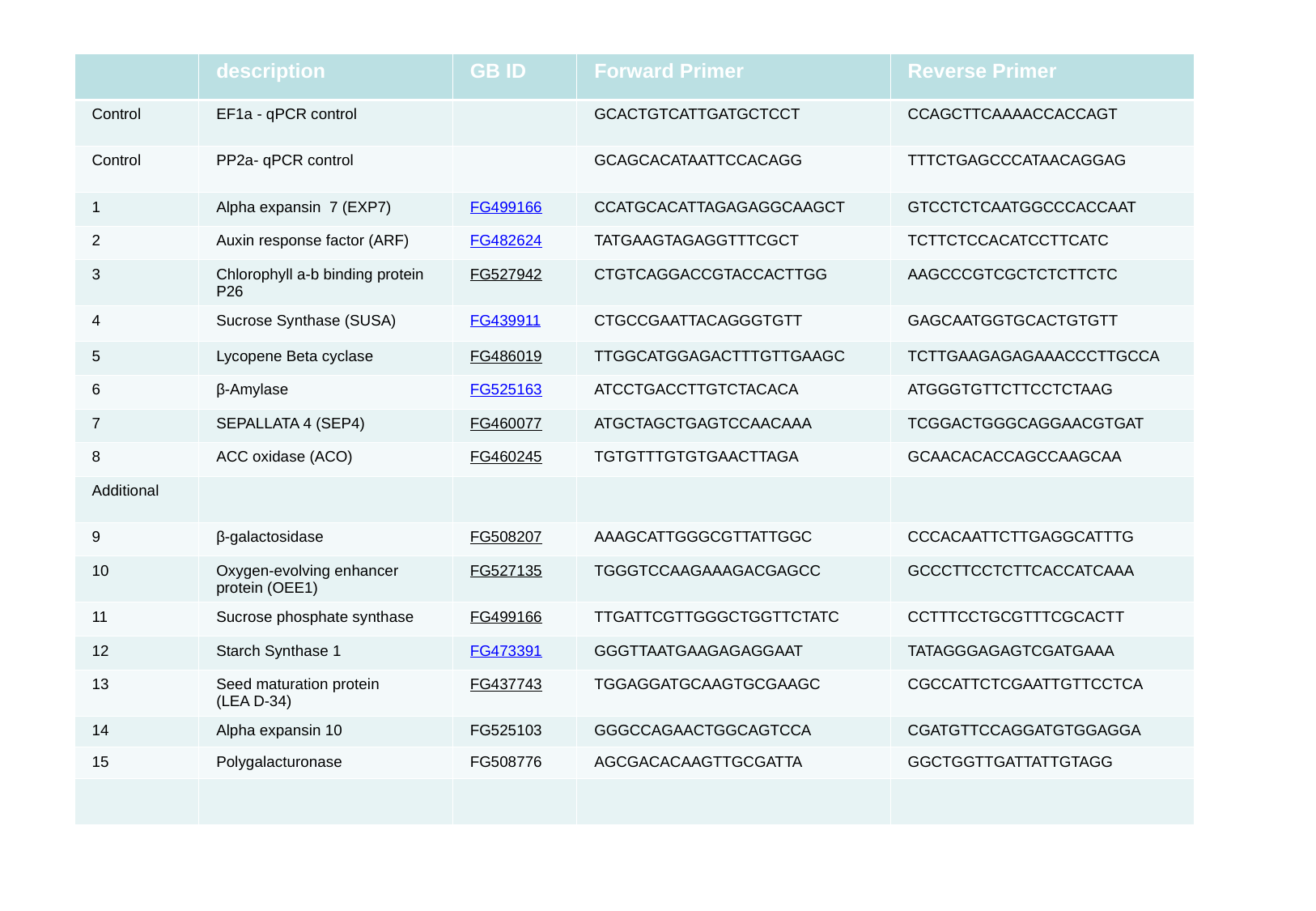

| | description | GB ID | Forward Primer | Reverse Primer |
| --- | --- | --- | --- | --- |
| Control | EF1a - qPCR control | | GCACTGTCATTGATGCTCCT | CCAGCTTCAAAACCACCAGT |
| Control | PP2a- qPCR control | | GCAGCACATAATTCCACAGG | TTTCTGAGCCCATAACAGGAG |
| 1 | Alpha expansin 7 (EXP7) | FG499166 | CCATGCACATTAGAGAGGCAAGCT | GTCCTCTCAATGGCCCACCAAT |
| 2 | Auxin response factor (ARF) | FG482624 | TATGAAGTAGAGGTTTCGCT | TCTTCTCCACATCCTTCATC |
| 3 | Chlorophyll a-b binding protein P26 | FG527942 | CTGTCAGGACCGTACCACTTGG | AAGCCCGTCGCTCTCTTCTC |
| 4 | Sucrose Synthase (SUSA) | FG439911 | CTGCCGAATTACAGGGTGTT | GAGCAATGGTGCACTGTGTT |
| 5 | Lycopene Beta cyclase | FG486019 | TTGGCATGGAGACTTTGTTGAAGC | TCTTGAAGAGAGAAACCCTTGCCA |
| 6 | β-Amylase | FG525163 | ATCCTGACCTTGTCTACACA | ATGGGTGTTCTTCCTCTAAG |
| 7 | SEPALLATA 4 (SEP4) | FG460077 | ATGCTAGCTGAGTCCAACAAA | TCGGACTGGGCAGGAACGTGAT |
| 8 | ACC oxidase (ACO) | FG460245 | TGTGTTTGTGTGAACTTAGA | GCAACACACCAGCCAAGCAA |
| Additional | | | | |
| 9 | β-galactosidase | FG508207 | AAAGCATTGGGCGTTATTGGC | CCCACAATTCTTGAGGCATTTG |
| 10 | Oxygen-evolving enhancer protein (OEE1) | FG527135 | TGGGTCCAAGAAAGACGAGCC | GCCCTTCCTCTTCACCATCAAA |
| 11 | Sucrose phosphate synthase | FG499166 | TTGATTCGTTGGGCTGGTTCTATC | CCTTTCCTGCGTTTCGCACTT |
| 12 | Starch Synthase 1 | FG473391 | GGGTTAATGAAGAGAGGAAT | TATAGGGAGAGTCGATGAAA |
| 13 | Seed maturation protein (LEA D-34) | FG437743 | TGGAGGATGCAAGTGCGAAGC | CGCCATTCTCGAATTGTTCCTCA |
| 14 | Alpha expansin 10 | FG525103 | GGGCCAGAACTGGCAGTCCA | CGATGTTCCAGGATGTGGAGGA |
| 15 | Polygalacturonase | FG508776 | AGCGACACAAGTTGCGATTA | GGCTGGTTGATTATTGTAGG |
| | | | | |
